# Female infertility and long-term cardiovascular risk: a systematic review and meta-analysis

**DOI:** 10.1007/s12020-025-04543-x

**Published:** 2026-02-11

**Authors:** Christina Polymeropoulou, Nikoletta Mili, Eleni Armeni, Elina Siliogka, Areti Augoulea, Irene Lambrinoudaki

**Affiliations:** 1 Second Department of Obstetrics and Gynaecology, National and Kapodistrian University of Athens, Aretaieio Hospital, Athens, Greece; 2 Department of Applied Health Sciences, School of Health Sciences, College of Medicine and Health, University of Birmingham, Birmingham, UK

**Keywords:** Cardiovascular disease, Infertility, Assisted reproduction techniques, Coronary heart disease, Stroke

## Abstract

**Objective:**

To investigate the association between a personal history of infertility and the risk for future cardiovascular disease (CVD) and related manifestations like coronary heart disease (CHD), cerebrovascular disease and heart failure in women. We also considered the potential influence of exposure to assisted reproductive technology (ART) on the risk of future CVD.

**Methods:**

This systematic review and meta-analysis of 22 studies, registered in PROSPERO (CRD42023420300), followed PRISMA guidelines. Pooled odds ratios (OR) and hazard ratios (HR) were calculated using Mantel-Haenszel and inverse variance methods, while heterogeneity was evaluated using I². Sensitivity analyses were performed to explore the impact of study design.

**Results:**

We observed that women with infertility compared to controls had a higher risk of incident CVD (infertile vs controls: 178,828 vs 3,398,781; HR = 1.14, 95% CI 1.12-1.16; I² = 89%), CHD (HR=1.17, 95% CI 1.12-1.23; I² = 0%), and cerebrovascular events (HR=1.16, 95% CI 1.11-1.21; I²=73%). Exposure to ART was associated with a higher risk of overall CVD across five studies (HR = 1.17, 95% CI 1.11–1.24; I² = 96%) and with increased incident stroke across four studies (HR = 1.12, 95% CI 1.03–1.23; I² = 60%), whereas no significant association was observed with CHD. However, some of the estimates were heterogenous, which limits certainty and causal inference.

**Discussion:**

Women with infertility compared with controls had higher pooled estimates for incident CVD, CHD and cerebrovascular events. However, these estimates were heterogeneous (I² up to 99%), which limits certainty and precludes causal inference.

## Introduction

Cardiovascular disease (CVD) includes a range of disorders affecting the heart and blood vessels, such as coronary artery disease (CHD), hypertension, and stroke [1**–**3]. CVD manifestations are the leading cause of death among women worldwide, representing about 35% of female fatalities [4]. CHD affects 250 million globally, followed by peripheral arterial disease (110 million), stroke (94 million), and atrial fibrillation (53 million), highlighting the widespread impact of distinct cardiovascular conditions [5]. Considering the differences in the prevalence rates of CVD between men and women, the scientific focus started exploring differences in risk factors of CVD between the two genders [6]. Adverse pregnancy outcomes like preeclampsia, gestational diabetes, and preterm delivery are increasingly recognized as factors for cardiometabolic risk in women [6]. These conditions may accelerate vascular aging, highlighting the importance of including obstetrical history in cardiovascular assessments and considering such events as “metabolic syndrome equivalents” [7].

On the other hand, the possible link between a personal history of infertility and CVD has received more limited attention. Infertility is defined as the inability to achieve pregnancy after 12 months or more of regular, unprotected sexual intercourse. Among couples of reproductive age, infertility is prevalent at rates of 12.6–17.5% and appears to be more frequent in regions such as America, Africa, the Pacific and Europe [3]. Despite the variable prevalence between regions, evidence on the possible link between infertility and socioeconomic status is still conflicting [8]. Unfortunately, infertility services are often inaccessible due to social stigma, high costs, and limited healthcare availability, despite affecting around one in six people worldwide [9].

Infertility is increasingly recognized as a marker for heightened cardiovascular risk in women due to shared pathophysiological pathways such as chronic inflammation, endothelial dysfunction, and adverse metabolic profiles [10**]**. Women with conditions linked to infertility, like polycystic ovary syndrome (PCOS) and metabolic syndrome, frequently demonstrate insulin resistance, dyslipidaemia, and hypertension, all of which compound long-term cardiovascular risk [10**]**. Emerging evidence also indicates that the use of assisted reproductive technologies (ART) is associated with an elevated risk of subsequent CVD. The causal nature of this association remains to be elucidated; prevailing hypotheses implicate the increased incidence of hypertensive disorders of pregnancy and gestational diabetes mellitus observed among women conceiving via ART as potential mediating pathways [11**]**. These findings underscore the importance of integrating [12**]** early cardiometabolic screening and preventive strategies into the care of women with a history of infertility [13**]**.

The possible link between subfertility and CVD events has received attention by one meta-analysis that explored the possible association between fertility therapy and long-term cardiovascular outcomes [14**]**. This meta-analysis could not highlight any associations between the use of fertility therapy and long-term cardiovascular outcomes after assessing studies characterised by substantial heterogeneity [14**]**. A recent umbrella review reported that the risk for composite CVD was higher for women with a personal history of stillbirth when compared to women with no history of stillbirth. The same risk was comparable between women with a history of miscarriage and those who did not experience a miscarriage [15**]**. Furthermore, others reported that apart from the traditional risk factors, the risk for future CHD is also predicted by the number of stillbirths and the number of miscarriages, indicating a possible interaction between a personal history of infertility and the risk for CHD [16**]**.

This systematic review and meta-analysis aims to explore the potential link between female infertility and the likelihood of developing CVD in later life. Specifically, the study aims to ascertain whether women who experience infertility face a higher risk of cardiovascular events, such as CHD, stroke, or heart failure, compared to those without a history of subfertility, infertility treatment or both.

## Methods

### Study outcomes

Eligibility criteria were observational studies, namely cross-sectional, case-control and prospective cohort studies that investigated women with a history of subfertility and the possible association with incident cardiovascular and cerebrovascular disease.

### Search strategy

This review was conducted and reported according to the Preferred Reporting Items for Systematic Reviews and Meta-Analyses (PRISMA) guidelines [17**]**. The study was registered in PROSPERO (registration number CRD42023420300). All eligible studies were assessed using the following databases: Medline, Embase, Web of Science, Scopus, and the Cochrane Library. We also performed citation searching of the included studies and key reviews to identify additional eligible publications. Terms referring to the exposure, namely outcomes cardiovascular disease (CVD) and coronary heart disease (CHD) were combined, generating the following search string: (((female) AND ((fertility)OR(infertility) OR (subfertility) OR (sterility))) AND ((cardiovascular disease) OR (coronary disease) OR (myocardial infarction) OR (atherosclerosis) OR (cerebrovascular disease) OR (stroke) OR (ischemic heart disease) OR (congestive heart failure)).

Studies pursued were those that investigated the association between a personal history of infertility or subfertility and the experience of CVD events, including fatal or non-fatal CHD, cerebrovascular disease (hemorrhagic and ischemic stroke), congestive heart failure (CHF) or sudden death.

The data search was conducted independently by three researchers (ES, CP and NM), blinded to each other’s decisions, who assessed the electronic databases and selected individual studies fulfilling inclusion criteria. The results were discussed at the first time frame set for data collection (November 2024) and updated at the second time frame (September 2025). Potential disagreements were discussed with two researchers (EA and IL) to ensure all eligible studies were added.

All potentially eligible studies were downloaded and imported into the *Excel* software tool for deduplication. The three independent researchers screened the abstracts and titles of the identified records, and non-relevant content were excluded. The remaining articles were retrieved in full text to evaluate eligibility, whereas the reason for exclusion was recorded for the studies not considered eligible.

### Search strategy and study selection

The first step of data extraction was performed using a predesigned Micsosoft Excel^®^ spreadsheet, which was piloted using five eligible studies and edited as needed for improvement. All references retrieved from the above-named databases and/or manual screen were imported into Covidence® for deduplication, screening, and data management. Potentially overlapping datasets (e.g. repeated analyses of the same cohort at different timepoints) were identified within Covidence by cross-checking study names, recruitment periods, and registry IDs. When overlapping cohorts were detected, only the most complete and up-to-date publication was included; overlapping cohorts were explicitly excluded.

Searches were limited to studies published in English and used pre-specified search strings combining terms for exposure (female infertility, subfertility, sterility) and outcomes of interest. The primary outcome of this review was the association between a personal history of infertility and the risk of incident CVD, including CHD, cerebrovascular events (hemorrhagic or ischemic stroke), and CHF. The secondary outcome was the same set of cardiovascular endpoints (incident CVD, CHD, cerebrovascular events and CHF) among women with infertility who were exposed to ART compared with those not exposed. For studies reporting multiple or composite outcomes, we pre-specified that the most clinically relevant or clearly defined cardiovascular outcome would be extracted; composite outcomes were only used when specific outcomes could not be disaggregated. This ensured consistency across studies and minimized double counting of events.

The following data were extracted from each study: i) first author’s name and year of publication; ii) study cohort, design and sample size; iii) mean age of participants at the time of recruitment to the study; iv) mean time of follow up; v) definition of infertility or subfertility; vi) definition of CVD outcomes assessed; vii) number of women with history of infertility or subfertility; viii) number of women without infertility; ix) type of infertility treatment; x) the number of CVD events in women stratified according to the presence or absence of a history of infertility or subfertility or stratified according to the intake of infertility treatment or lack there-off; xi) incident risk rates for the development of CVD or CVD-related events, stratified according to the personal history of infertility and/or subfertility or according to the intake of infertility treatment, in studies not providing absolute numbers.

In case of missing data, the principal study investigators were contacted for unreported data or to provide additional details. Potential disagreements were discussed to ensure the inclusion of relevant data. Data extraction and management took place using the *Review Manager* (*RevMan* computer program), version 5.4 software (Copenhagen: The Nordic Cochrane Centre, The Cochrane Collaboration, 2014).

### Risk of bias assessment

The quality of incorporated studies was assessed using the Newcastle-Ottawa scale (NOS). This system explores the quality of studies based on the criteria of participant selection (maximum four starts), comparability of the study groups (maximum two stars) and the assessment of exposure or outcome (maximum three stars). This risk of bias assessment tool has been validated for case-control and cohort studies, whereas a modified version of the scale is available for cross-sectional studies [18**]**. The quality of the selected studies was assessed according to the maximum scores achieved on the NOS scale.

### Statistical analysis

All study estimates were converted to the log hazard ratio (log-HR) before pooling to ensure a common metric across studies. Between-study heterogeneity was assessed using Cochran’s χ² test and quantified with the I² statistic, with values of 40–60 % and >60 % interpreted as moderate and high heterogeneity, respectively. Between-study variance (τ²) was estimated with DerSimonian–Laird and verified in sensitivity analyses with REML. We prespecified random-effects models when conceptual or clinical heterogeneity was present or when I² ≥ 40%; fixed-effect models were used otherwise. Heterogeneity was assessed by χ² and quantified by I² (<60% moderate; >60% high). Robustness was examined via leave-one-out analyses and by excluding high-risk-of-bias studies.

For studies reporting absolute numbers of events, associations between women with a history of infertility and those without (controls) and the subsequent risk of cardiovascular or cerebrovascular events were expressed as odds ratios (ORs) with 95 % confidence intervals (CIs) using the Mantel–Haenszel method. For studies reporting only incidence rates, pooled hazard ratios (pHR) were calculated using the inverse-variance method, comparing women with a history of infertility to those without, as well as women exposed to assisted reproductive technology (ART) to those unexposed.

Pre-specified subgroup analyses by study design were conducted. A two-sided p-value <0.05 was considered statistically significant. All analyses and risk-of-bias assessments were performed in Review Manager (RevMan) version 5.4 (Copenhagen: The Nordic Cochrane Centre, The Cochrane Collaboration, 2014).

## Results

### Qualitative analysis

**Fig. 1:**
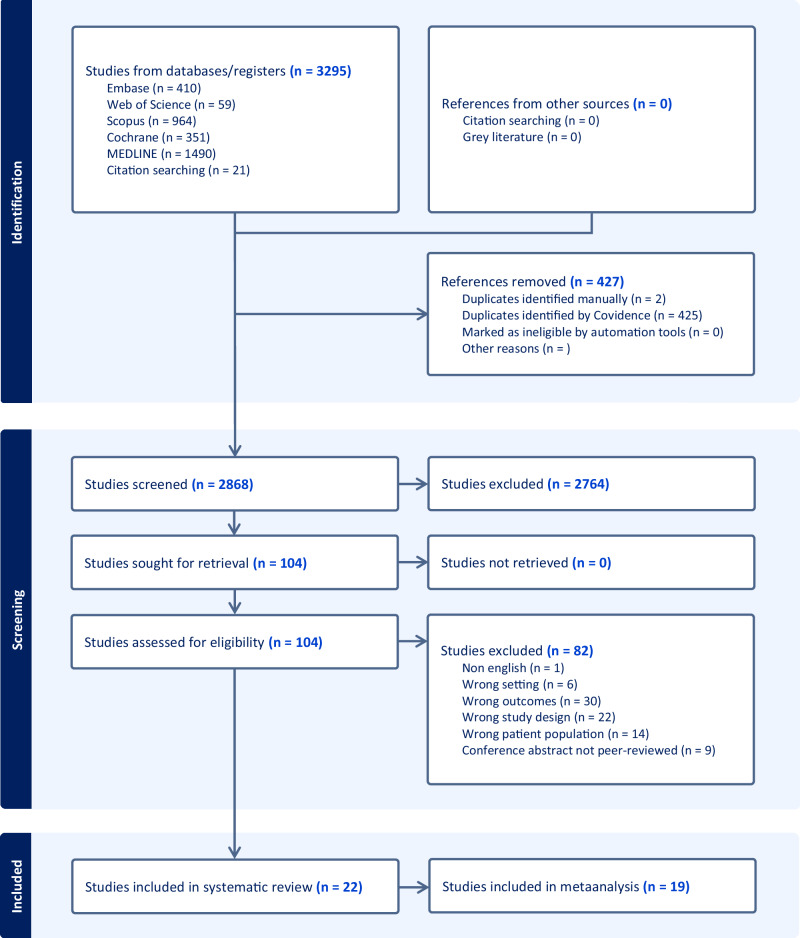
PRISMA 2020 flow diagram of study selection: Infertility and CVD risk

Twenty-two studies were eligible and included in the systematic review [13,19–39] (flowchart in Fig. 1). The descriptive characteristics of the eligible studies describing cardiovascular or cerebrovascular events in women with a prior diagnosis of infertility vs non-infertile controls as well as in women exposed to assisted reproduction techniques (ART) vs those not exposed to ART are shown in Table 1. The quality of the studies, as assessed by the NOS, is presented in Table 2. All studies had sufficient quality as evaluated using the NOS criteria.

**Table 1:** Descriptive characteristics of studies included in systematic review

First author, year	Study design	Number of participants	Mean follow up (years)	Infertility / Subfertility definition	CVD outcomes	Mean age at study participation (years)	
						Infertility	No infertility
Udell, 2013	Prospective cohort	1,186,753	9.7 (IQR 4.6-14.0)	Fertility therapy use within 2 years before delivery, from health insurance claims	Death or hospitalization for a major adverse cardiovascular event; (nonfatal coronary ischemia, stroke, transient ischemic attack, thromboembolism, or HF)	29 (25–33)	34 (31–36)
Westerlund, 2014	Prospective cohort	140,458	Infertility: 8.6 (SD 4.6) Controls: 8.6 (SD 4.9)	IVF treatment recorded in national IVF and birth registers	Stroke, CHD	33.3 (4.0)	33.4 (3.9)
Ben-Yaakov, 2016	Retrospective cohort	99,291	11 (SD 7)	Hospital records of fertility treatments (IVF, ovulation induction, IUI)	Hospitalization due to cardiovascular reasons or diagnostic procedures	30.9 (6)	28.7 (6)
Ge, 2018	Retrospective cohort	23,550	-	Use of ovulation-inducing medications (clomiphene, gonadotropins, etc.)	Ischemic stroke, and cardiovascular disease	47.9 (11.22)	32.7 (7.4)
Bungum, 2019	Prospective cohort	87,221	8.9	IVF treatment in national registry	Incident hospitalization due to cvd (cerebrovascular disease and ischemic heart diseases)	32.6 (4.8)	31.9 (4.8)
Murugappan, 2019	Retrospective cohort	3,192,690	Infertility 3.8 (SD 3.3) No infertility 3.9 (SD 3.3)	Infertility diagnosis and treatment identified via ICD and CPT codes	Cerebrovascular disease and ischemic heart diseases	34 (5.7)	32.7 (7.4)
Gleason, 2019	Cross-sectional	744	4 years	Self-reported infertility (≥12 months trying without conception)	Congestive HF, coronary heart disease, heart attack, or stroke	39.7 (11.44)	
Cairncross, 2020	Prospective cohort	2,809	10	Self-reported infertility (≥12 months trying without conception)	Any atherosclerotic cvd event (stroke, angina, myocardial infarction)	45.7 (2.7)	45.8 (2.7)
Liang, 2022	Individual participant pooled analysis of eight prospective cohort studies.	94,286	13 - 9.4 (non fatal stroke- fatal stroke)	Self-reported infertility (>12 months to conceive)	Non-fatal or fatal stroke	–	
Lau, 2022	Prospective cohort	38,528	15 (IQR 8–20)	Self-reported infertility (>12 months to conceive)	HF	63 (7)	–
Murugappan, 2022	Prospective cohort	158,787	Infertility: 19.3 (SD 5.1) No infertility: 19.3 (SD 5.2)	Self-reported infertility (>12 months trying)	First-time ASCVD event (clinical myocardial infarction, coronary revascularization, ischemic stroke, peripheral arterial disease, carotid artery disease, and death from cardiovascular disease)	63.2 (7.4)	63.2 (7.2)
Skara,2022	Prospective cohort	31,629	14 (SD 9)	Self-reported subfertility (>12 months trying); partner fertility inferred via birth registry	Stroke, CHD, angina, myocardial infarction, any CVD	47.9 (12.1)	45.1 (10.2)
Sachdev, 2023	Retrospective cohort	31,339,991	12 months postpartum	Infertility and ART identified from ICD codes during delivery hospitalization	Hospitalization for non-fatal stroke	32.1	27.7
Farland, 2023	Prospective cohort	103,729	28 years	Self-reported infertility (≥12 months trying); cause-specific (ovulatory, tubal, endometriosis, unexplained)	myocardial infarction, stroke (cerebrovascular accident or transient ischemic attack), coronary artery bypass grafting/ angioplasty/stent	34.9 (4.6)	34.8 (4.7)
Magnus, 2024	Prospective cohort	2,496,441	11 (IQR 5-18)	Registered delivery as conceived with the use of ART vs. without	Any registration of ischemic heart disease (including myocardial infarction), cerebrovascular disease (including stroke), cardiomyopathy, HF, pulmonary embolism, and deep vein thrombosis .	29.1 (4.9)	33.8 (4.7)
Tomic, 2024	Retrospective cohort	27,262	11.8	ART (IVF/ICSI/FET) recorded in national registers	Any CVD hospitalizations (ischaemic heart diseases, sudden cardiac death, atrial fibrillation and flutter)	35	36
Tang, 2024	Prospective cohort	75,778	16.84 (IQR 14.77–18.78)	Registered for fertility treatment (IVF clinic records)	Stroke mortality	62 (IQR 57, 67)	62 (IQR 58, 67)
Yamada, 2024	Retrospective cohort	31,339,991	12 months postpartum	Self-reported infertility (>12 months to conceive)	Hospitalization due to heart disease (including ischemic heart disease, atherosclerotic heart disease, cardiomyopathy, hypertensive disease, HF, and cardiac dysrhythmias)	–	–
Wei, 2024	Retrospective cohort	1,001,593	11 years	Infertility treatment identified via ICD codes	Cardiovascular hospitalizations	–	–

*IQR*, interquartile range; *CVD*, cardiovascular disease; *CHD*, coronary heart disease; *HF*, heart failure; *IVF*, in vitro fertilization; *SD*, standard deviation; *IUI*, intrauterine insemination; *ICD*, International Classification of Diseases; *CPT*, Current Procedural Terminology; *ASCVD*, atherosclerotic cardiovascular disease; *ART*, assisted reproductive technology; *ICSI*, intracytoplasmic sperm injection; *FET*, frozen embryo transfer; *MAR*, medically assisted reproduction

**Table 2:** Newcastle–Ottawa Scale (NOS) Quality Assessment of Cohort Studies

Study (Year)	Design	Selection (0–4★)	Comparability (0–2★)	Outcome (0–3★)	Total (0–9★)
Udell (2013)	Population cohort	★★★★	★★	★★★	9
Westerlund (2014)	Registry cohort	★★★★	★★	★★	8
Ben-Yaakov (2016)	Retrospective cohort	★★★★	★★	★★	8
Ge (2018)	Registry cohort	★★★★	★★	★★	8
Bungum (2019)	Registry cohort	★★★★	★	★★★	7
Murugappan (2019)	Retrospective cohort	★★★	★★	★★★	8
Gleason (2019)	Cross-sectional	–	–	–	not NOS-applicable
Cairncross (2020)	Prospective cohort	★★★	★★	★★★	7
Liang (2022)	Consortium cohort	★★★★	★★	★★★	9
Lau (2022)	Prospective cohort	★★★★	★★	★★★	9
Murugappan (2022)	Prospective cohort	★★★★	★★	★★★	9
Skara (2022)	Prospective cohort	★★★★	★★	★★★	9
Sachdev (2023)	Retrospective cohort	★★★★	★★	★★	8
Farland (2023)	Prospective cohort	★★★★	★★	★★★	9
Magnus (2024)	Registry cohort	★★★★	★★	★★★	9
Tomic (2024)	Retrospective cohort	★★★	★★	★★	7
Tang (2024)	Prospective cohort	★★★★	★★	★★★	9
Yamada (2024)	Retrospective cohort	★★★★	★★	★★	8
Wei, 2024	Retrospective cohort	★★★★	★★	★★★	9

Our cohort included one cross-sectional study, namely the analysis of women retrieved from the NHANES survey (National Health and Nutrition Examination Survey) evaluated a total of 744 women (117 with a history of infertility and 627 without) and reported that the risk for cardiovascular events is increased (odds ratio, OR 1.41; 95% CI: 0.96 to 2.25) in women with compared to those without infertility [23**]**.

The incidence rates of cardiovascular events in women with versus without a personal history of infertility have been reported in six studies. Notably, only one retrospective cohort study [29**]** reported a significantly higher risk of cardiovascular events in women with a prior history of infertility compared with those without infertility (absolute incidence: 21.91 vs. 17.98 per 1000 person-years; HR 1.22, 95% CI 1.19–1.26). In contrast, five prospective studies [13, 20, 21, 28, 32] did not demonstrate a significant association between a personal history of infertility and future cardiovascular risk. Of note, in two prospective cohort studies, the absolute incidence rates were consistently higher among women with a history of infertility compared with controls (ranging from 3.64 to 10.42 vs. 3.20 to 8.89 per 1000 person-years, respectively). However, the relative risks were similar between the two groups, with HRs of 1.09 (95% CI 0.99–1.20) and 1.08 (95% CI 0.99–1.18) [13, 28].

Regarding the future risk for CHD events, four prospective studies [13, 20, 28, 32] and one retrospective study [29**]** have examined this association, with two of the prospective studies also reporting absolute incidence rates [13, 32]. In these prospective studies, the risk for CHD was higher among women with a personal history of infertility when compared with those without such a history (2.45 per 1000 person-years and 73.1 per 100,000 person-years vs 2.15 per 1000 person-years and 57.6 per 100,000 person-years, respectively). However, one prospective study did not demonstrate a significant association between a personal history of infertility and future CHD risk (HR 1.02, 95% CI: 0.82–1.26) [20**]**. The single retrospective study likewise reported a higher absolute incidence of CHD among women with a history of infertility compared with controls (3.8 vs 3.19 per 1000 person-years) [29**]**. The relative risk estimates were heterogenous: two studies reported elevated higher age-adjusted hazards for CHD (HR 1.16–1.19) [13, 32]

Regarding the future risk for cerebrovascular disease events, three prospective studies [25, 32, 37] and one retrospective study [29] reported an increased risk among women with a history of infertility compared with those without such a history. Absolute incidence rates for stroke were either comparable between women with and without infertility (31.6 vs 31.7 per 100,000 person-years) [13] or marginally higher among women with a history of infertility (1.19 vs 1.01 per 1000 person-years) [32**]**. Conversely, the retrospective study [29**]** reported a clearly higher absolute risk of stroke in women with a history of infertility compared with controls (2.05 vs 1.57 per 1000 person-years). However, two prospective studies found no significant difference in the risk of future cerebrovascular events between women with and without a personal history of infertility [13, 20].

The risk of CHF was examined in a single study [24**]** which reported a higher age-adjusted hazard of CHF in women with a history of infertility compared with controls (HR 1.16, 95% CI: 1.04 to 1.30).

A number of studies reported the risk for incident CVD comparing women with a prior exposure to ART as opposed to those not exposed to ART. The incident risk for future CVD in women with prior exposure to ART vs those not exposed to ART was evaluated by two prospective studies [27, 34] and three retrospective studies [22, 36, 39], with pooled absolute event rates ranging from 1.03 to 1.93 per 1000 person-years among ART-exposed women versus 1.17 to 42.28 per 1000 person-years among non-exposed comparators, which did not demonstrate any significant difference and indicated between study variability. The relative hazard for CVD events was found to be comparable between women exposed to ART and controls in the two prospective studies [27, 34, 39]; only one retrospective study indicated higher hazard for CVD in women exposed to ART vs controls, with HR of 1.76 (95% CI: 1.59-1.95) [36**]** A third prospective study compared CVD mortality rates between 33,520 women who underwent in vitro fertilisation and 10,629 women who did not receive fertility treatment [38**]**. The study reported lower CVD mortality rates among women who underwent fertility treatment (absolute CVD mortality 0.14 per 1000 person-years vs 0.35 per 1000 person-years in controls), with a standardized mortality rate of 0.41 (95% CI: 0.32–0.53) [38**]**.

Similarly, no significant difference was found with regard to the future risk for CHD events in women with prior exposure to ART as opposed to those not exposed to ART, according to the results of three prospective [27, 34, 35], with absolute CHD incidence reported between 0.03 and 53.2 per 100,000 person-years in ART-exposed women and 0.02 to 35.5 per 100,000 person-years in non-exposed women. The relative incidence rates also varied, with three studies [27, 34, 35] reporting comparable hazard between women exposed to ART and controls and only one study [36] reporting a HR of 1.73 (95% CI: 1.12-2.66). The three retrospective studies [33, 36, 39] reported inconsistent results with either hazard of 1.73 [36**]** or non-significant risk for CHD [33, 39].

The risk for future cerebrovascular events was found to be significantly higher in women with a prior exposure to ART as opposed to those not exposed to ART (three prospective [27, 34, 35], three retrospective studies [22, 31, 39]), with absolute rates in prospective studies approaching 0.39-42.8 per 100,000 person-years in ART-exposed women versus 0.32–35 per 100,000 person-years in comparators. Considering the relative incidence, one prospective study [34**]** and one retrospective studies [31**]** described a higher hazard for stroke of 1.76–2.14 for women exposed to ART vs controls, while the second retrospective study showed non-significant risk [39**]**

Finally, no difference was described in the risk for future CHF between women exposed to ART and those not exposed, according to the results of two prospective studies [27, 34], with absolute CHF incidence of 9.3–18.6 per 100,000 person-years in women exposed to ART compared to 9.1–14.6 per 100,000 person-years, yet there was no difference in measures of relative risk. No difference in the risk for CHF was reported by retrospective studies [33, 36, 39].

### Quantitative analysis

A total of 19 studies were eligible to be included in the meta-analysis.

**Table 3:** **a**Studies comparing CVD in women with infertility vs. controls. **b** Studies comparing coronary heart disease and heart failure events in women with infertility vs. controls. **c** Studies comparing stroke in women with infertility vs. controls

a									
First author, year	Study design	Sample size	Women with infertility	Controls	Events in Women with infertility (n)	Events in controls (n)	Absolute incidence in Women with infertility	Absolute incidence in controls	Relative effect measures (HR or OR, 95% CI)
**Gleason, 2019**	Cross-sectional	744	117	627	5	17	—	—	OR 1.41 (0.96-2.25)
**Murugappan 2019**	Retrospective cohort	3,035,291	60,528	2,974,763	4,775	199,150	21.91 /1,000 PY	17.98 /1,000 PY	HR 1.22 (1.19-1.26)
**Bungum, 2019**	Prospective cohort	87,221	53,806	33,415	686	250	—	—	HR 1.04 (0.90-1.20)
**Cairncross, 2021**	Prospective cohort	2,809	695	2,114	—	—	—	—	HR 0.80 (0.54-1.19)
**Skara, 2022**	Prospective cohort	31,629	5,321	26,308	—	—	3.64 /1,000 PY	3.20 /1,000 PY	HR 1.09 (0.99-1.20)^a^
**Murugappan, 2022 (WHI)**	Prospective cohort	158,787	25,933	132,854	4,000	19,860	—	—	HR 1.03 (0.99-1.07)
**Farland, 2023**	Prospective cohort	103,729	28,611	75,118	761	1,489	104.2 /100,000 PY	88.9 /100,000 PY	HR 1.08 (0.99–1.18)^a^

b									
First author, year	Study design	Sample size	Women with infertility	Controls	Events in Women with infertility (n)	Events in controls (n)	Absolute incidence in Women with infertility	Absolute incidence in controls	Relative effect measures (HR or OR, 95% CI)
***Coronary heart disease events***									
**Bungum, 2019**	Prospective cohort	87,221	53,806	33,415	321	111	—	—	HR 1.02 (0.82-1.26)
**Murugappan 2019**	Retrospective cohort	3,169,036	63,797	3,105,239	917	38,481	3.80 /1,000 PY	3.19 /1,000 PY	HR 1.19 (1.12-1.27)
**Skara, 2022**	Prospective cohort	31,629	5,321	26,308	—	—	2.45 /1,000 PY	2.15 /1,000 PY	HR 1.19 (1.06-1.33)^a^
**Murugappan, 2022 (WHI)**	Prospective cohort	158,787	25,933	132,854	1,084 (clinical MI) 1,523 (cardiac procedure)	5,330 (clinical MI) 7,507 (cardiac procedure)	—	—	—
**Farland, 2023**	Prospective cohort	103,729	28,611	75,118	534	966	73.1 /100,000 PY	57.6 /100,000 PY	HR 1.16 (1.05–1.29)^a^
***Heart Failure***									
**Lau, 2022**	Prospective cohort	38,528	5,399	33,129	—	—	—	—	HR 1.16 (1.04-1.30)^a^

c									
First author, year	Study design	Sample size	Women with infertility	Controls	Events in Women with infertility (n)	Events in controls (n)	Absolute incidence in Women with infertility	Absolute incidence in controls	Relative effect measures (HR or RR, 95% CI)
**Bungum, 2019**	Prospective cohort	87,221	53,806	33,415	384	146	—	—	HR 1.04 (0.86-1.27)
**Murugappan 2019**	Retrospective cohort	3,184,783	64,135	3,120,648	502	19,135	2.05 /1,000 PY	1.57 /1,000 PY	HR 1.32 (1.2-1.44)
**Skara, 2022**	Prospective cohort	31,629	5,321	26,308	—	—	1.19 /1,000 PY	1.01 /1,000 PY	HR 1.19 (1.02-1.39)^a^
**Liang, 2022**	Individual participant pooled analysis of eight prospective cohort studies	94,187 (data on non-fatal stroke)	16,211	77,976	453 (non-fatal)	2,292 (non-fatal)	—	—	HR 1.13 (1.05-1.21) (non-fatal)
		39,656 (data on fatal stroke)	5,168	34,488	27(fatal)	177 (fatal)	—	—	—
**Murugappan, 2022 (WHI)**	Prospective cohort	158,787	25,933	132,854	863	4,420	—	—	—
**Farland, 2023**	Prospective cohort	103,729	28,611	75,118	231	531	31.6 /100,000 PY	31.7 /100,000 PY	HR 0.92 (0.79–1.07)^a^
**Tang, 2024**	Prospective cohort	75,778	11,004	64,774	196	958	—	—	HR 1.21 (1.04-1.41)

^a^HR adjusted for age

*CVD*, cardiovascular disease; *CHD*, coronary heart disease; *HF*, heart failure; *HR*, hazard ratio; *CI*, confidence interval; *PY*, person-years; *MI*, myocardial infarction

#### Women with a history of infertility vs non-infertility

##### Studies reporting the absolute number of events and/or odds ratios (Table 3a–c)

**Fig. 2:**
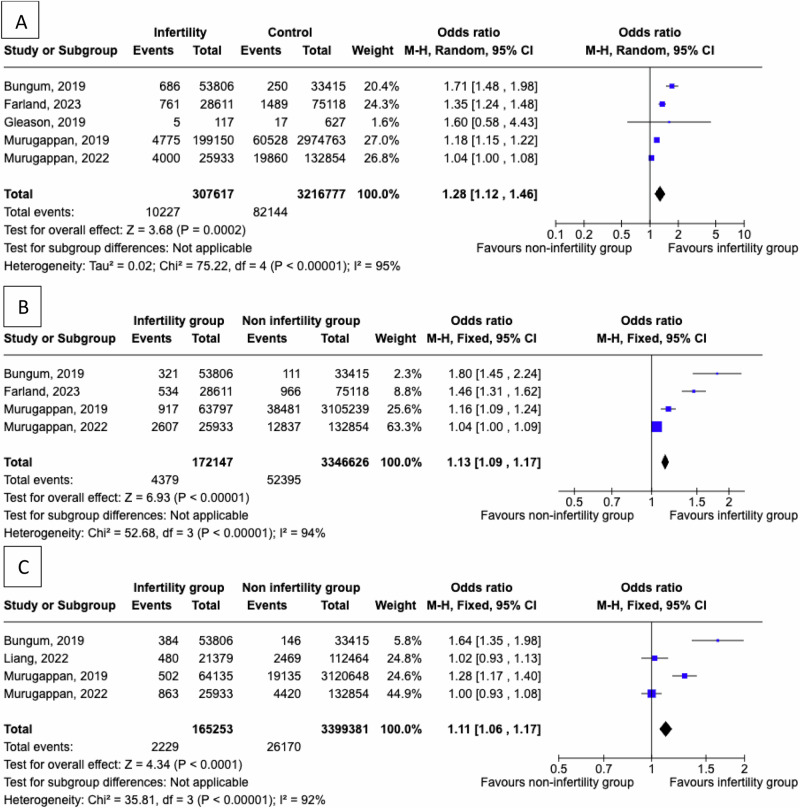
Women with a personal history of infertility were compared to women without infertility to evaluate the absolute risk of **A**) any cardiovascular events, **B**) coronary heart disease events, **C**) cerebrovascular events

The risk for CVD events in women with a personal history of infertility as opposed to control women is presented in Fig. 2A. We found a significantly higher risk for CVD events (pooled OR = 1.28, 95% CI: 1.12 to 1.46) in an analysis of five studies [13, 20, 23, 28, 29] with 307,617 women with a history of infertility and 3,216,777 controls. The analysis was characterised by significant heterogeneity (I^2^ = 95%).

The risk for prevalent CHD in women with a personal history of infertility as opposed to control women is presented in Fig. 2B. We found a significantly higher risk for prevalent CHD events (pooled OR = 1.13, 95% CI: 1.09 to 1.17) in an analysis of six studies [13, 20, 28, 29, 33, 35] with 172,147 infertile women and 3,346,626 controls. The analysis was characterised by significant heterogeneity (I^2^ = 94%).

The risk for cerebrovascular events in women with a personal history of infertility vs controls is presented in Fig. 2C. We found a significantly higher risk for cerebrovascular events (pooled OR = 1.11, 95% CI: 1.06 to 1.17) in an analysis of five studies [20, 25, 28, 29, 35] with 165,253 infertile women and 3,399,381 controls. The analysis was characterised by significant heterogeneity (I^2^ = 92%).

##### Studies reporting hazard ratios

**Fig. 3:**
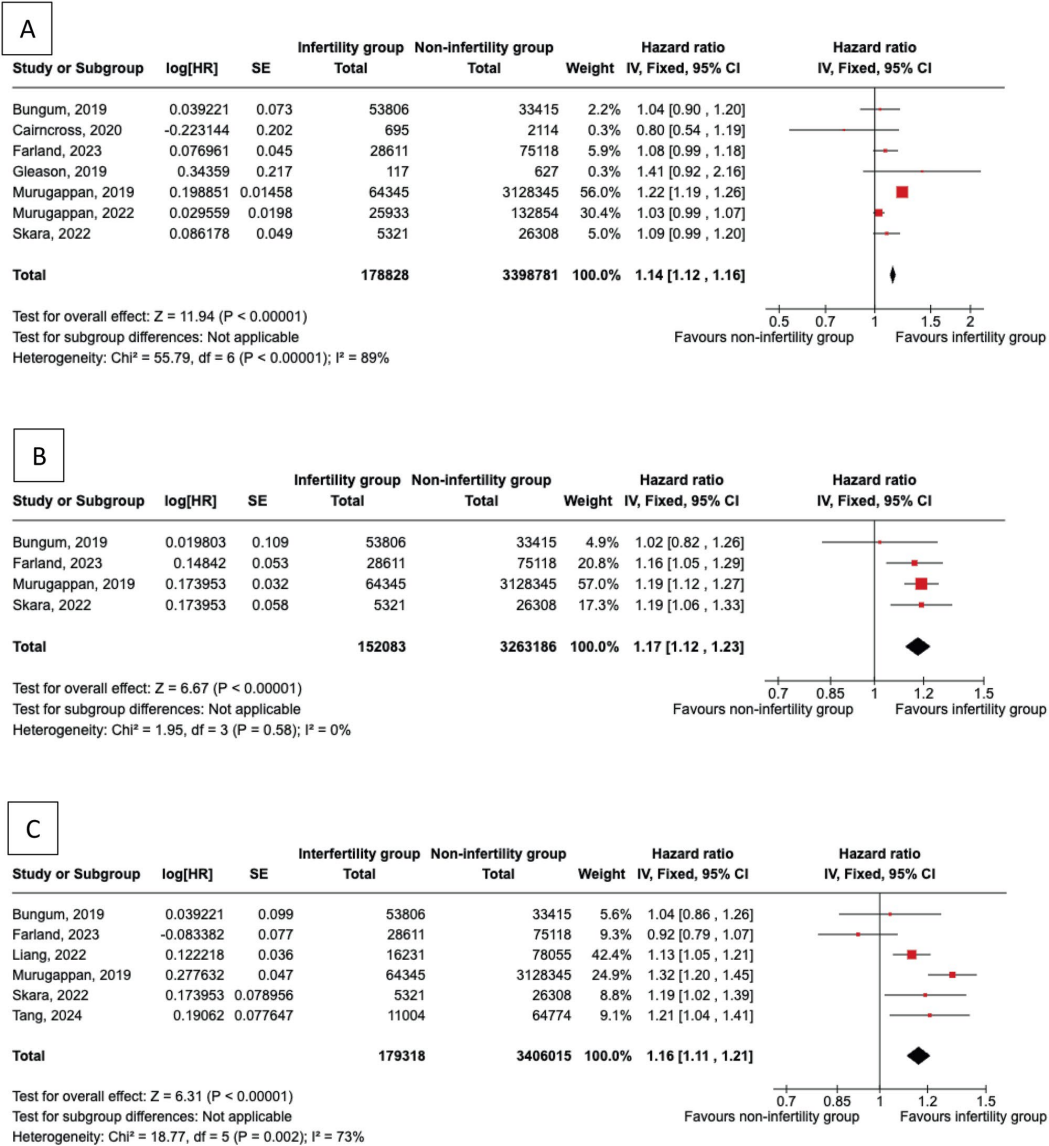
Women with a personal history of infertility were compared to women without infertility to evaluate the incidence of **A**) any cardiovascular events, **B**) coronary heart disease events, **C**) cerebrovascular events

We evaluated the incidence for CVD, CHD and cerebrovascular events in women with a personal history of infertility and controls. We found a significantly higher risk for future CVD events (pHR = 1.14, 95% CI: 1.12 to 1.16) in an analysis of seven studies [13, 20, 21, 23, 28, 29, 32] that included 178,828 infertile women and 3,398,781 controls, as presented in Fig. 3A. The analysis was characterised by significant heterogeneity (I^2^ = 89%).

With regards to CHD events, we found a significantly higher risk for women with a history of infertility vs controls (pHR = 1.17, 95% CI: 1.12 to 1.23), assessing four studies [13, 20, 29, 32], with a total of 152,083 women with a history of infertility and 3,263,186 controls, as presented in Fig. 3B. The analysis was characterised by low heterogeneity (I^2^ = 0%).

With regards to the risk for future cerebrovascular events, we found a significantly higher risk for women with a history of infertility vs controls (pHR = 1.16, 95% CI: 1.11 to 1.21; moderate heterogeneity I^2^ = 73%), assessing six studies [13, 20, 25, 29, 32, 37], with a total of 179,318 women with infertility and 3,406,015 controls, as presented in Fig. 3C.

**Table 4:** **a** Studies comparing CVD in women who used MAR vs. controls. **b** Studies comparing CHD in women who used MAR vs. controls. **c** Studies comparing HF in women who used MAR vs. controls. **d** Studies comparing Stroke in women who used MAR vs. controls

a									
First author, year	Study design	Sample size	MAR	Controls	Events in Women with infertility (n)	Events in Controls (n)	Absolute incidence in Women with infertility	Absolute incidence in controls	Relative effect measures (HR or RR, 95% CI)
**Udell, 2013**	Prospective cohort	186,753	6,979	179,774	44	12,730	1.03/1000 PY	1.17/1000 PY	HR 0.96 (0.72–1.29)
**Westerlund, 2014**	Prospective cohort	140,458	23,498	116,960	–	–	–	–	–
**Ben-Yaakov, 2016**	Retrospective cohort	99,291	4,153	95,138	117	3,139	–	–	–
**Ge, 2018**	Retrospective cohort	23,550	4,710	18,840	–	–	–	–	HR 0.84 (0.75–0.95)
**Tomic, 2024**	Retrospective cohort	27,262	24,131	3131	6,268	523	–	–	RR 0.92 (0.81–1.04)
**Magnus, 2024**	Prospective cohort	2,496,441	97,474	2,398,967	1,303	42,685	1.89/1,000 PY	1.52/1,000 PY	HR 1.04 (0.99–1.10)^a^
**Yamada, 2024**	Retrospective cohort	31,339,991	287,813	31,052,178	1582	110,106	1.52/1,000 PY	42.28/1,000 PY	HR 1.76 (1.59–1.95)
**Wei, 2024**	Retrospective cohort	1,001,593	22887	978,706	207	10,447	1.93/1,000 PY	1.84/1,000 PY	HR 1.15 (0.98–1.34)

b									
First author, year	Study design	Sample size	MAR	Controls	Events in Women with infertility (n)	Events in Controls (n)	Absolute incidence in Women with infertility	Absolute incidence in controls	Relative effect measures (HR or RR, 95% CI)
**Udell, 2013**	Prospective cohort	186,753	6,979	179,774	–	–	14.0/100,000 PY	16.7/100,000 PY	HR 1.23 (0.55–2.74)
**Westerlund, 2014**	Prospective cohort	140,458	23,498	116,960	23	185	11.4/100,000 PY	18.5/100,000 PY	HR 0.65 (0.42–1.00)
**Tomic, 2024**	Retrospective cohort	27,262	24,131	3131	348	14	–	–	RR 1.13 (0.66 – 1.93)
**Magnus, 2024**	Prospective cohort	2,496,441	97,474	2,398,967	370	10,019	53.2/100,000 PY	35.5/100,000 PY	HR 1.03 (0.93–1.15)^a^
**Yamada, 2024**	Retrospective cohort	31,339,991	287,813	31,052,178	63	4908	0.03/100,000 PY	0.02/1,000 PY	HR 1.73 (1.12–2.66)
**Wei, 2024**	Retrospective cohort	1,001,593	22,887	978,706	11	480	10.2/100,000 PY	8.5/100,000 PY	HR 1.41 (0.70–2.83)

c									
First author, year	Study design	Sample size	MAR	Controls	Events in Women with infertility (n)	Events in Controls (n)	Absolute incidence in Women with infertility	Absolute incidence in controls	Relative effect measures (HR or RR, 95% CI)
**Udell, 2013**	Prospective cohort	186,753	6,979	179,774	–	–	18.6 /100,000 PY	14.6 /100,000 PY	HR 1.13 (0.56–2.26)
**Tomic, 2024**	Retrospective cohort	27,262	24,131	3131	297	14	–	–	RR 1.16 (0.68–1.99)
**Magnus, 2024**	Prospective cohort	2,496,441	97,474	2,398,967	65	2,559	9.3/100,000 PY	9.1/100,000 PY	HR 0.83 (0.64–1.06)^a^
**Yamada, 2024**	Retrospective cohort	31,339,991	287,813	31,052,178	198	21,902	287.2/100,000 PY	2.7/100,000 PY	HR 1.07 (0.85–1.34)
**Wei, 2024**	Retrospective cohort	1,001,593	22,887	978,706	9	504	8.4/100,000 PY	8.8/100,000 PY	HR 0.98 (0.47–2.03)

d									
First author, year	Study design	Sample size	MAR	Controls	Events in Women with infertility (n)	Events in Controls (n)	Absolute incidence in Women with infertility	Absolute incidence in controls	Relative effect measures (HR or RR, 95% CI)
**Udell, 2013**	Prospective cohort	186,753	6,979	179,774	–	–	16.3/100,000 PY	8.5/100,000 PY	HR 2.14 (1.02–4.50)
**Westerlund, 2014**	Prospective cohort	140,458	23,498	116,960	79	319	0.39/100,000 PY	0.32 /100,000 PY	HR 1.24 (0.97–1.58)
**Ge, 2018**	Retrospective cohort	23,550	4,710	18,840	–	–	–	–	HR 0.85 (0.70–1.03)
**Sachdev, 2023**	Retrospective cohort	31,339,991	28,7813	31,052,178	–	–	37/100,000 PY	29 /100,000PY	HR 1.76 (1.25–2.48)
**Magnus, 2024**	Prospective cohort	2,496,441	97,474	2,398,967	298	9,892	42.8/100,000 PY	35/100,000 PY	HR 1.05 (0.94–1.18)^a^
**Wei, 2024**	Retrospective cohort	1,001,593	22,887	978,706	22	1059	20.4/100,000 PY	18.8/100,000 PY	HR 1.18 (0.71–1.97)

^a^HR adjusted for age, calender year

*CVD*, cardiovascular disease; *CHD*, coronary heart disease; *HF*, heart failure; *HR*, hazard ratio; *CI*, confidence interval; *PY*, person-years; *PM*, person-months

#### Women with infertility: exposed vs non-exposed to ART

##### Studies reporting absolute numbers and/or odds ratios (Table 4a–c)

**Fig. 4:**
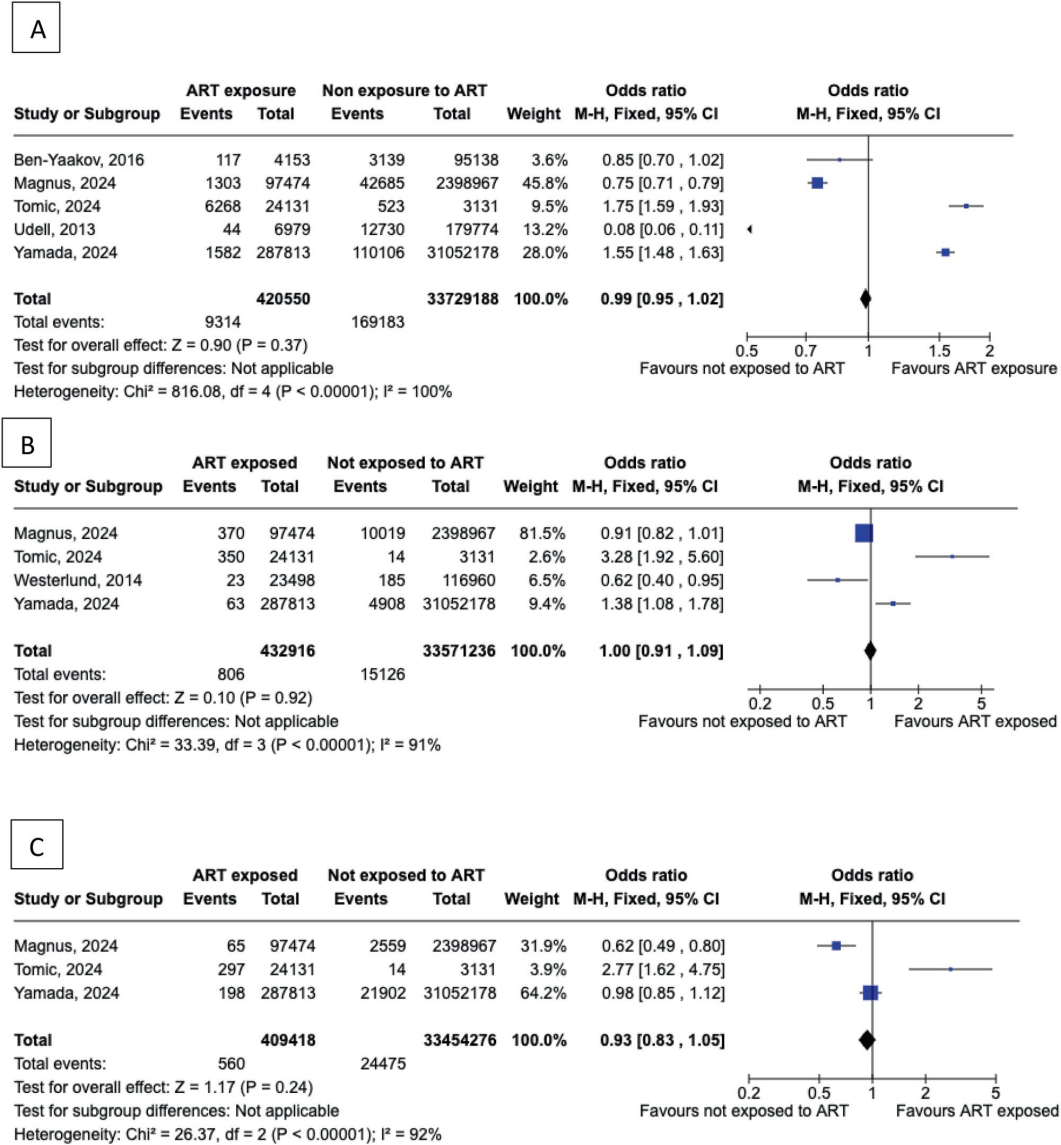
Women exposed to assisted reproduction techniques were compared to women not exposed to fertility treatment to evaluate the absolute risk of **A**) any cardiovascular events, **B**) coronary heart disease events, **C**) cerebrovascular events

We found no significant difference in the risk of CVD events between women exposed to ART and those not exposed, evaluating five studies [19, 27, 33, 34, 36] with 420,550 women exposed to ART and 33,729,188 controls (pooled OR = 0.99, 95% CI: 0.95 to 1.02; I^2^ = 100%, Fig. 4A). Similarly, prior exposure to ART was not associated with a difference in the risk for CHD events compared to lack of exposure, after the assessment of four studies [27, 33, 35, 36], consisting of 432,916 women exposed to ART and 33,571,236 controls (pooled OR = 1.00, 95% CI: 0.91 to 1.09; I^2^ = 91%, Fig. 4B). Finally, we found no significant difference in the risk of CHF comparing women exposed to ART vs those not exposed to ART, after the evaluation of three studies, which included 409,418 women exposed to ART and 33,454,276 controls (pooled OR = 0.93, 95% CI: 0.83 to 1.05; I^2^ = 92%; Fig. 4C).

##### Studies reporting hazard ratios

**Fig. 5:**
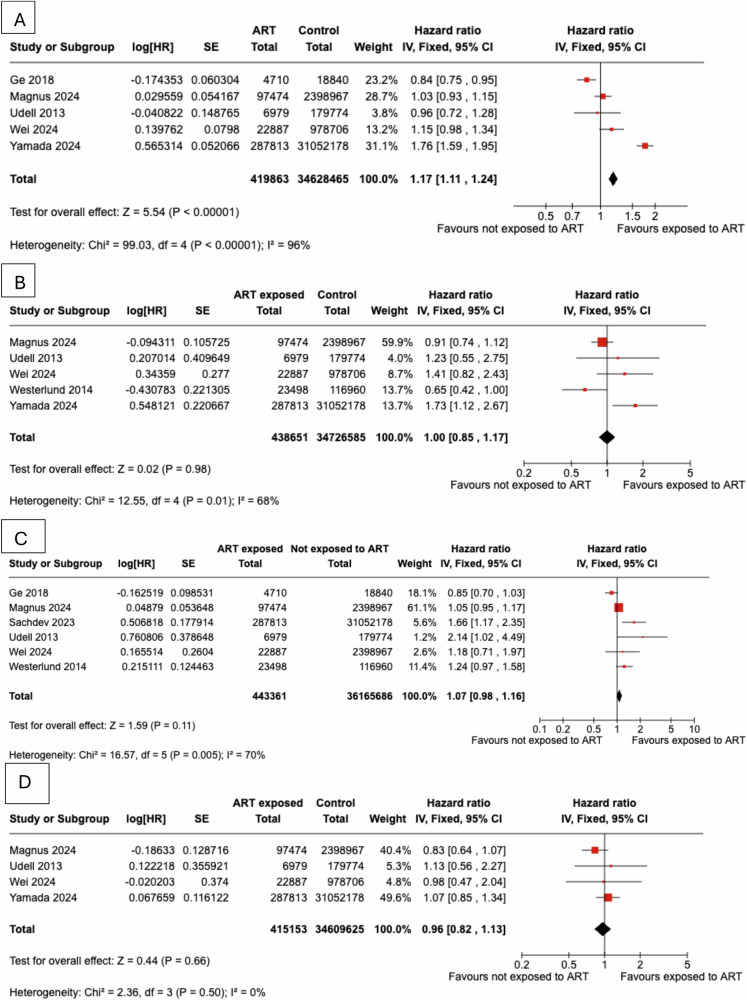
Women exposed to assisted reproduction techniques were compared to women not exposed to fertility treatment to evaluate the incidence of **A**) any cardiovascular events, **B**) coronary heart disease events, **C**) cerebrovascular events, **D**) heart failure

We compared the incidence of CVD events between women exposed to ART and those not exposed in a total of five studies [22, 27, 34, 36, 39], which comprise a total of 419,863 women exposed to ART and 34,628,465 controls, as presented in Fig. 5A. The incidence of CVD events was higher in women exposed to ART vs controls (pHR = 1.17, 95% CI: 1.11 to 1.24), yet the studies were characterised by substantial heterogeneity (I^2^ = 96%). Sequential leave-one-out analyses demonstrated that exclusion of any single study did not materially alter the pooled effect estimates or the degree of heterogeneity for studies evaluating cardiovascular disease risk in women with a history of ART compared with controls.

We compared the incidence of CHD events between women exposed to ART and those not exposed, in a total of four studies [27, 34–36, 39], which comprise a total of 438,651 women exposed to ART and 34,726,585 non-exposed, as presented in Fig. 5B. The incidence of CHD events did not differ between women exposed to ART vs controls (pHR = 1.00, 95% CI: 0.85 to 1.17), and the studies were characterized by moderate heterogeneity (I^2^=68%). Sequential leave-one-out analyses showed that removing any single study did not materially alter the pooled effect estimates or the degree of heterogeneity for studies evaluating coronary heart disease risk in women with a history of ART compared with controls

We compared the incidence of cerebrovascular events between women exposed to ART and those not exposed in a total of five studies [22, 27, 34, 35], comprising 443,361 women exposed to ART and 36,165,686 non-exposed, as presented in Fig. 5C. The incidence of cerebrovascular events did not differ between women exposed to ART vs controls (pHR = 1.07, 95% CI: 0.98 to 1.16), and the studies were characterised by moderate heterogeneity (I^2^ = 70%). Omission of the [22] study yielded a statistically significant association between ART use and future risk for stroke, with a pHR of 1.12 (95% CI 1.03–1.23; I^2^ = 60%) among 438,651 women exposed to ART compared with 36,146,846 controls.

The incidence of CHF after exposure to ART vs women not exposed to ART, was evaluated in a total of three prospective studies [27, 34, 36, 39], which comprise a total of 415,153 women exposed to ART and 34,609,625 non-exposed, as presented in Fig. 5D. The incidence of CHF did not differ between women exposed and those non-exposed to ART (pHR = 0.96, 95% CI: 0.82 to 1.13). The studies were characterized by low heterogeneity (I^2^ = 0%).

### Sensitivity analysis

#### Analysis by study design for studies comparing women with infertility vs no-infertility

##### Prospective studies

The risk for incident CVD was evaluated in five prospective studies [13, 20, 21, 28, 32], which comprised a sample of 114,366 infertile women and 269,809 controls. Women with a history of infertility had a higher risk for a future CVD event (pHR = 1.04, 95% CI: 1.01 to 1.08; Supplemental Fig. 1a). These studies were characterized by no heterogeneity (I^2^ = 0%).

The risk for future CHD events was evaluated in three studies [13, 20, 32], which comprised a sample of 87,738 infertile and 134,841 non-infertile women. The risk for future CHD events was higher in women with a personal history of infertility vs controls (pHR = 1.15, 95% CI: 1.07 to 1.24; Supplemental Fig. 1b). The analysis was characterised by no heterogeneity (I^2^ = 0%).

The risk for future cerebrovascular events was evaluated in five prospective studies [13, 20, 25, 32, 37], which comprised a sample of 114,973 infertile and 277,670 control women. Women with a history of infertility had higher risk for stroke in comparison to control women (pHR = 1.11, 95% CI: 1.05 to 1.17; Supplemental Fig. 1c). The analysis was characterized by moderate heterogeneity (I^2^ = 54%).

##### Retrospective studies

Only one retrospective study was included in our meta-analysis, hence further assessment was not possible.

#### Analysis by study design for studies comparing women exposed vs non-exposed to ART

##### Prospective studies

The risk for incident CHD events in women stratified by exposure to ART was evaluated in three studies [27, 34, 35], which comprised a sample of 127,951 women exposed to ART and 2,695,701 not exposed to ART. The analysis showed that the risk for future CHD events did not differ according to the exposure to ART (pHR = 0.87; 95% CI: 0.73 to 1.04; Supplemental Fig. 2a). The studies were characterized by moderate heterogeneity (I^2^ = 24%).

The risk for incident cerebrovascular events in women stratified by exposure to ART was evaluated in three studies [27, 34, 35], which comprised a sample of 127,951 women exposed to ART and 2,695,701 not exposed to ART. The analysis showed that the risk for future cerebrovascular events did not differ according to the exposure to ART (pHR = 1.09, 95% CI: 0.99 to 1.20; Supplemental Fig. 2b). The studies were characterized by moderate heterogeneity (I^2^ = 58%).

##### Retrospective studies

The risk for incident CVD events in women stratified by their exposure to ART was evaluated in three studies [27, 36, 39], which comprised a sample of 315,410 women exposed to ART and 32,049,724 women not exposed to ART. The risk for future CVD events was significantly higher in those exposed to ART vs controls (pHR = 1.55, 95% CI: 0.82 to 2.93; Supplemental Fig. 2c). The studies were characterized by significant heterogeneity (I^2^ = 99%). Sequential leave-one-out analyses showed that removing any single study did not materially alter the pooled effect estimates or the degree of heterogeneity for studies evaluating coronary heart disease risk in women with a history of ART compared with controls.

## Discussion

This systematic review and meta-analysis of 22 studies, encompassing nearly 180,000 women with infertility and more than 3 million controls, examined the association between female infertility and subsequent CVD. We observed higher prevalence and incidence of CVD—including CHD and cerebrovascular events—among women with a history of infertility. Sensitivity analyses indicated that the relative risk of future cardiovascular events was greater among women who experienced infertility earlier in their reproductive life than among those with later onset. Exposure to ART was associated with an increased incidence of CVD; however, the magnitude of this association was modest (pHR typically ~1.1–1.2) and its true impact remains uncertain due to substantial heterogeneity across studies, likely reflecting differences in study design and ART protocols. These findings underscore the importance of contextualising effect sizes and emphasising clinical relevance while avoiding overstatement of their potential public health implications.

To our knowledge, this is the first meta-analysis to investigate the possible role of a personal history of infertility as marker for cardiovascular events. These results were confirmed even after sensitivity analysis for the study design. Despite the lack of high-quality evidence on the link between a history of infertility and long-term CVD, studies on nulliparous women are in agreement with our findings. A meta-analysis of 18 studies, including 2,813,481 participants with no life birth, demonstrated a 1.19-times higher pooled relative risk of all-cause mortality compared to participants with a prior life birth [40**]**. Moreover, the incident risk for cardiovascular death in nulliparous women was estimated as 2.43-times higher than in parous women [41**]**.

Comparing the possible association between exposure to ART and future cardiovascular events, we found a higher incident risk of any CVD event but no difference in the risk of CHD or stroke or CHF, while the studies were characterised with significant heterogeneity. A previous meta-analysis focused solely on the possible effect of exposure to ART with regard to future cardiovascular risk [14**]** and reported a similar risk of cardiac events but a trend toward higher risk of stroke between women exposed to ART and those not exposed to ART, following an assessment of six observational studies with less specifically defined cardiovascular outcomes and significant heterogeneity. Partly supporting our findings, another meta-analysis of 16 cohort studies and 2 case control studies with 7,808,501 women, reported inconsistent and inconclusive associations between a personal history of interfertility and stroke [42**]**.

In evaluating the association between infertility and future cardiovascular disease, several potential sources of confounding and reverse causation warrant explicit consideration. Conditions such as PCOS, metabolic syndrome and endometriosis are highly prevalent among women with infertility and are themselves associated with adverse cardiometabolic profiles [43, 44]. These disorders may therefore act as intermediates or common antecedents linking infertility and CVD, rather than infertility per se being causally related to CVD risk. Residual confounding by unmeasured lifestyle and treatment factors (e.g. hormonal therapies, pregnancy complications) may further influence observed associations [45**]**. Future studies should incorporate rigorous adjustment for these co-morbidities and explore mediation pathways to disentangle the independent contribution of infertility from that of underlying conditions

While infertility and cardiovascular disease manifest differently, they share common risk factors and underlying mechanisms. Recent evidence highlighted a link between the atherogenic index of plasma and infertility, even after control of significant cardiovascular risk factors [46**]**. The inflammatory process, which tends to present with conditions like endometriosis or polycystic ovary syndrome, contributes to endothelial dysfunction [47, 48] and atherosclerosis [49**]**. Oxidative stress and increased levels of reactive oxygen species are interacting with the tissues of the female reproductive tract, causing inflammation and damage to the endometrium, oocyte and ovarian function, and fallopian tubes [50**]**.

The results of this study highlight the need for clinicians to be encouraged to prioritize modifiable CVD risk factors, such as body mass index, blood pressure, lipid levels, and glucose, in women experiencing infertility, especially those with PCOS or early-onset infertility. Evidence on the cost-effectiveness of incorporating reproductive history (e.g., infertility/PCOS) directly into CVD risk algorithms is limited [51**]**. However, recent UK work (QR4) shows that including pregnancy complications such as pre-eclampsia improves risk prediction, supporting the principle of integrating reproductive history into risk assessment [52**]**. Moreover, economic evaluations in women with hypertensive disorders of pregnancy demonstrate that postpartum risk-factor screening and remote blood-pressure management are cost-effective or cost-saving, suggesting likely clinical and economic value for targeted pathways informed by reproductive history [12**]**. Collectively, these data support the plausibility that reproductive-history–informed CVD screening could offset future morbidity, treatment, and hospitalisation costs, strengthening the case for its inclusion in preventive care frameworks.

This meta-analysis presents several limitations. First, exposure misclassification is possible: infertility and ART exposure were largely self-reported or registry-based, and subfertility not reaching specialist care may be under-captured. Second, outcome ascertainment relied on administrative codes in many cohorts, risking under- or over-ascertainment of CVD events. Third, between-study heterogeneity was substantial for several outcomes, reflecting variation in infertility definitions, ART protocols, covariate adjustment, and follow-up duration, which ranged from a few years to decades. Fourth, residual and time-varying confounding (e.g., PCOS, endometriosis, obesity, smoking, pregnancy complications, cardioprotective medication) and reverse causation cannot be excluded. Fifth, combining minimally and fully adjusted HRs may introduce bias despite prespecified selection rules. Sixth, potential immortal-time and left-truncation biases in registry designs, along with competing risks in older cohorts, may influence incidence estimates. Finally, restriction to English-language publications may have introduced selection and publication bias and limited inclusion of informative non-English cohorts.

This meta-analysis suggests that a history of infertility may be associated with an increased risk of CVD in women, including coronary heart disease and stroke, particularly when infertility is diagnosed at a younger age. Although exposure to assisted reproductive technology ART appeared to increase overall CVD risk, no consistent associations were found for coronary or cerebrovascular outcomes specifically. Given the observational nature of the included studies and the substantial heterogeneity across populations, definitions, and outcome measures, these findings should be interpreted with caution. Further high-quality, longitudinal research is warranted to clarify causal pathways and to determine the long-term cardiovascular impact of ART.

## Supplementary information

Supplemental Files

## Data Availability

No datasets were generated or analysed during the current study.
