# Supplementary material for: Female infertility and long-term cardiovascular risk: a systematic review and meta-analysis
Source: Endocrine. 2026 Feb 11;91(1):70. doi: 10.1007/s12020-025-04543-x (PMC12894145; doi:10.1007/s12020-025-04543-x)

Supplemental Figure 1. Studies comparing women with infertility vs women without infertility to assess the incidence for a) cardiovascular disease events, b) coronary heart disease events, c) cerebrovascular events

A


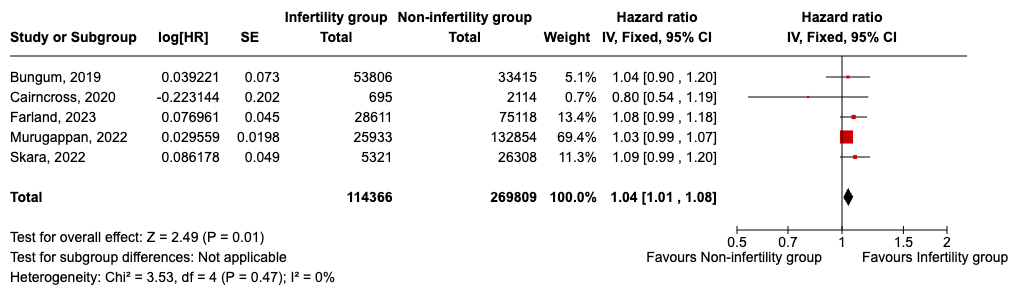


B/


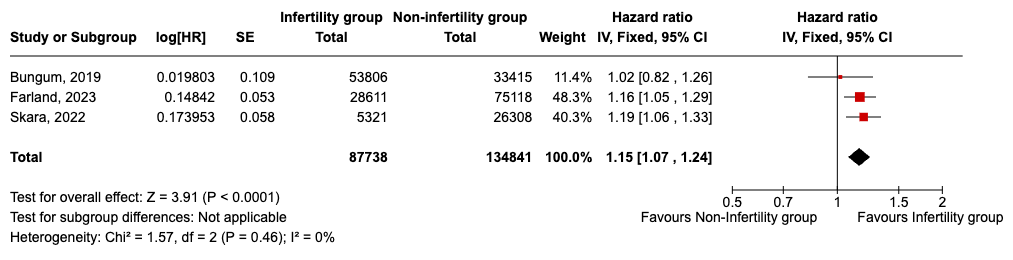


C/


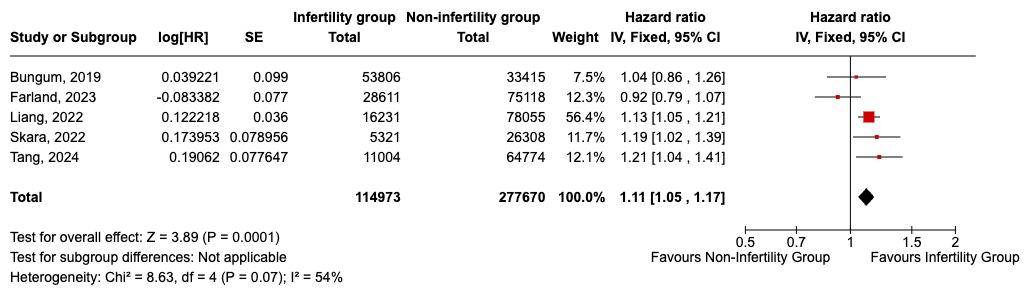

Supplement: Supplementary file 1 — Supplemental Files [file 12020_2025_4543_MOESM1_ESM.zip › Supplemental Files/Supplemental Figure 1.docx]
