# Supplementary material for: Female infertility and long-term cardiovascular risk: a systematic review and meta-analysis
Source: Endocrine. 2026 Feb 11;91(1):70. doi: 10.1007/s12020-025-04543-x (PMC12894145; doi:10.1007/s12020-025-04543-x)

**Supplemental Figure 2.** Analysis by study design for studies comparing women exposed vs non-exposed to assisted reproduction techniques: 1. Prospective studies assessing a) coronary heart disease events, b) cerebrovascular events; 2. Retrospective studies assessing cardiovascular events.

PROSPECTIVE STUDIES

A


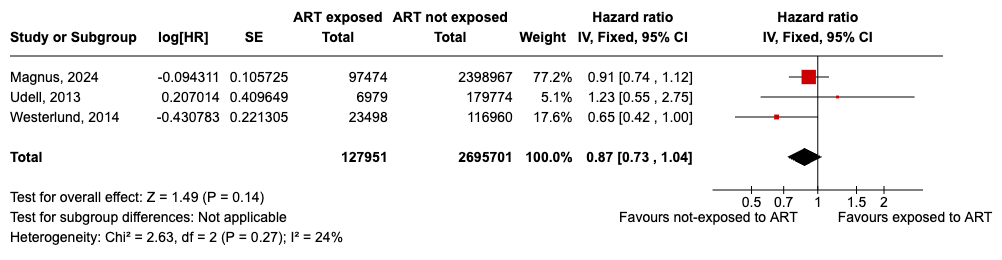


B/


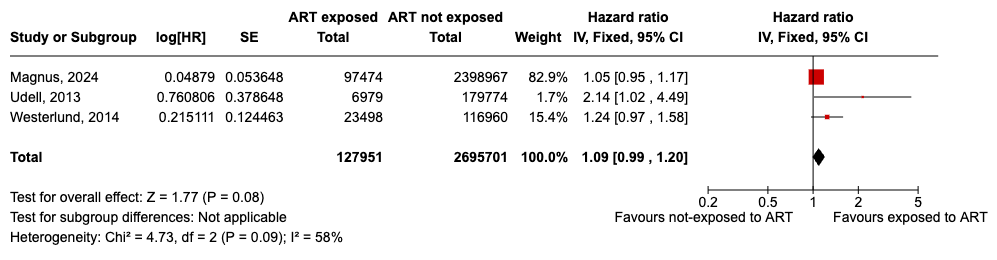


RETROSPECTIVE STUDIES

C/


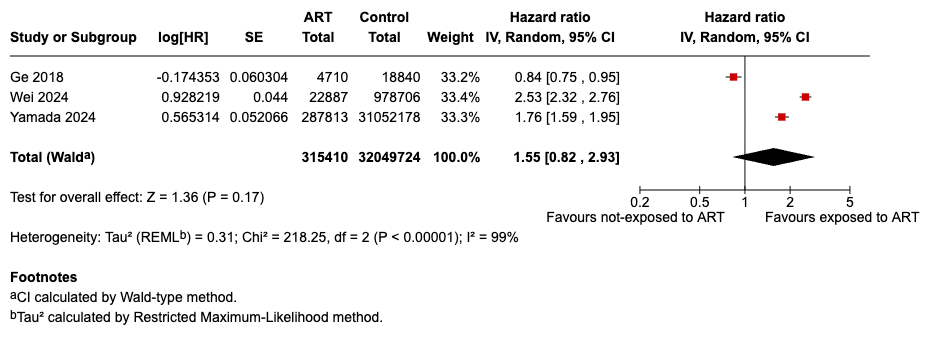

Supplement: Supplementary file 1 — Supplemental Files [file 12020_2025_4543_MOESM1_ESM.zip › Supplemental Files/Supplemental Figure 2.docx]
