# Supplementary material for: Female infertility and long-term cardiovascular risk: a systematic review and meta-analysis
Source: Endocrine. 2026 Feb 11;91(1):70. doi: 10.1007/s12020-025-04543-x (PMC12894145; doi:10.1007/s12020-025-04543-x)
Supplement: Supplementary file 1 — Supplemental Files [file 12020_2025_4543_MOESM1_ESM.zip › Supplemental Files/Supplementary Table 1.doc]

| **Supplementary table 1.** **Full-text articles excluded from data synthesis** | | | |
| --- | --- | --- | --- |
| **Study** | **Year** | **Reasons for exclusion** | **Number of studies** |
| Udell  Mancuso  Ge  Yao  Lockhart  Mahalingaiah  Kurabayashi  De Kat  Vent  Niemczyk  Verit  Heidari  Holzer  La Vecchia  Nichols  Parikh | 2017  2020  2019  2018  2017  2017  2016  2016  2016  2015  2014  2012  2007  1987  2024  2016 | Study design did not meet the inclusion criteria | 16 |
| Liang  Smith  Dayan | 2022  2021  2017 | Study type did not meet the inclusion criteria | 3 |
| Verit  Colditz | 2016  1987 | Patient population did not meet the inclusion criteria | 2 |
